# Editorial: Intramural Vascular Cells: Key Therapeutic Targets for Vascular Cognitive Impairment

**DOI:** 10.3389/fnagi.2020.615780

**Published:** 2020-10-30

**Authors:** Satoshi Saito, JoAnne McLaurin, Roxana Octavia Carare

**Affiliations:** ^1^Faculty of Medicine, University of Southampton, Southampton, United Kingdom; ^2^Department of Neurology, National Cerebral and Cardiovascular Center, Suita, Japan; ^3^Biological Sciences, Sunnybrook Research Institute, Toronto, ON, Canada

**Keywords:** smooth muscle cells (SMCs), pericytes, Alzheimer's disease, small vessel disease, vascular cognitive impairment, mural cells

Alzheimer's disease is the most common type of dementia characterized by neuropathological changes such as intracellular tau tangles and extracellular deposition of β-amyloid (Aβ) as plaques and cerebral amyloid angiopathy (CAA). As the important contribution of cerebrovascular dysfunction to Alzheimer's disease has been recently uncovered, research priorities have been gradually set for basic mechanisms and clinical evidence for the role of vascular factors in the pathogenesis of dementia (Sweeney et al., 2019). The main aim of the current Research Topic was to provide a comprehensive review of cerebrovascular mural cells as a novel therapeutic target for vascular cognitive impairment.

The mural cells include pericytes within the walls of capillaries and vascular smooth muscle cells (SMCs) in the tunica media of the cerebral arterioles and leptomeningeal arteries. SMCs are observed not only in the arteries but also in the veins. The ultrastructural characteristics of the arterial and venous SMCs are described in detail by Sharp et al.. The mural cells have been known as a contributor to architectural maintenance and contraction of vessels. Recent evidence further points to their critical roles in controlling brain homeostasis such as the management of cerebral blood flow (CBF) and the clearance of waste products from the brain.

CBF is critical for the delivery of oxygen and nutrients essential for neuronal and synaptic functioning and is constantly controlled by the mural cells in response to changes in transient neuronal activity, namely neurovascular coupling. Nevertheless, whether capillary pericytes or arterial SMCs initiate the regulation is controversial. This debate mainly stems from the different definitions of the pericytes. The mural cells on the proximal branches coming off penetrating arterioles are sometimes described as pericytes, but the same cells are named as SMCs in other reports, demonstrating the heterogeneity of this cell population (Grant et al., 2019). These cells are positioned at the transition between arterioles and capillaries and have shared some characteristics with pericytes and SMCs (Uemura et al.). In this topic, Zhou et al. showed the regional and large network cerebral dysfunction in a cohort of cerebral small vessel disease with gait disturbance by using functional MRI. Considering the crucial impact of neurovascular coupling on functional MRI, the pathogenic mechanism may be represented by degenerated mural cells in small vessel disease. Nelson et al. examined contractility of brain pericytes by employing a new optogenetic model developed by crossing pericyte-specific CreER mouse line with mice expressing channelrhodopsin-2. Excitation of channelrhodopsin-2 by 488 nm light resulted in pericyte contraction followed by constriction of the capillary leading to the reduction of regional blood flow. These results clearly demonstrate the pivotal roles of pericytes in the regulation of capillary blood flow.

Apart from their role in cerebral perfusion mural cells also contribute to the clearance of waste products from the brain. Small molecules are internalized by pericytes through receptor-mediated endocytosis or non-specific pinocytosis. In addition, mural cells are involved in the lymphatic drainage system of the brain. The central nervous system is devoid of classical lymph vessels. Instead, waste products from neurons and glia are cleared through the intramural periarterial drainage (IPAD) (Albargothy et al., 2018). Piotrowska et al. injected a fluorescent tracer into the entorhinal cortex of living and sacrificed rats and found that the tracer accumulated in the vascular outer and inner basement membrane in living rats, while it appeared to diffuse along the ventricles and hippocampal fibre tracts in sacrificed animals. The tracer was also observed in the cervical lymph nodes in living rats, but not in sacrificed, suggesting IPAD is an active clearance system driven by vital functions.

Soluble Aβ secreted from neurons diffuse and enter IPAD pathways within the basement membranes of capillaries continuing with the basement membranes surrounding SMCs of intracerebral arteries. Accumulating lines of evidence have shown that impaired IPAD is one of the potential mechanisms for the pathogenesis of Alzheimer's disease and CAA. IPAD flow rapidly moves toward the leptomeningeal arteries where the deposition of Aβ is prominent in CAA (Keable et al., 2016). Cerebrovascular Aβ accumulation eventually induce cerebral microbleeds as well as fatal ICH as shown by Saito et al.. Interestingly, Tsuji et al. reported that cilostazol, a phosphodiesterase 3 inhibitor, improved cognitive function in the Ts65Dn mouse, a model of Down syndrome. Patients with Down syndrome exhibit Aβ plaques and CAA, which is presumably due to the triplication of genes on human chromosome 21. Increased expression of the amyloid precursor protein gene would play a major role in the pathogenesis of CAA in Down syndrome, due to the overproduction of Aβ. Considering that cilostazol can facilitate IPAD (Maki et al., 2014), promotion of IPAD can be a therapeutic approach for Down syndrome as well as Alzheimer's disease.

Cerebral autosomal dominant arteriopathy with subcortical infarcts and leukoencephalopathy (CADASIL) and cerebral autosomal recessive arteriopathy with subcortical infarcts and leukoencephalopathy (CARASIL) are two of the representative hereditary small vessel diseases accompanied by degeneration of mural cells (Uemura et al., 2020). Both CADASIL and CARASIL are characterized by lacunar infarcts, cerebral microbleeds, white matter injury, and enlarged perivascular spaces. These changes are similar to the findings in patients with sporadic small vessel disease (Shindo, Ishikawa et al.), suggesting that understanding the pathogenesis of CADASIL and CARASIL is important for analysing sporadic small vessel disease (Mizuno et al.).

Within this series of papers, Panahi et al. demonstrated that gene and protein expression of GLUT2 and GLUT4 were altered in a cellular model and post-mortem brains of CADASIL patients, which was consistent with decreased CBF and glucose uptake in CADASIL patients. CADASIL has been considered a rare disease caused by the mutation of *NOTCH3* gene. Shindo, Tabei et al. estimated the prevalence rate as 1.20 to 3.58 per 100,000 adults based on the result of a nationwide survey in Japan. Nevertheless, Okada et al. reported 3.5% of Japanese patients presenting lacunar infarction had a *NOTCH3* mutation in genetic screening. The clinical phenotype of CADASIL is diverse, ranging from asymptomatic to severe; thus, *NOTCH3* mutations would be underdiagnosed, especially in patients who are currently diagnosed with sporadic small vessel disease.

Apart from proposing several pathogenic mechanisms for small vessel disease, this topic looks to the future with several therapeutic possibilities for vascular cognitive impairment. Levit et al. described the importance of antihypertensive treatment in Alzheimer's disease. They reported that white matter perivascular astrocytes had a central role in neurological vulnerability to hypertension by using transgenic rats overexpressing a pathogenic human amyloid precursor protein. Furthermore, Kato et al. proposed that transforming growth factor β signaling is a therapeutic target for protecting mural cell degeneration, as transgenic mice overexpressing transforming growth factor β1 exhibited decreased pericyte coverage and increased diameter of the capillaries. It is becoming apparent that mural cell degeneration contributes to the pathogenic processes of dementia including Alzheimer's disease. The pericytes and SMCs have become increasingly important as a therapeutic target and thus research on this topic is likely to accelerate in the future.

## Author Contributions

SS, JM, and RC contributed to conceptualization and writing. All authors contributed to the article and approved the submitted version.

## Conflict of Interest

The authors declare that the research was conducted in the absence of any commercial or financial relationships that could be construed as a potential conflict of interest.
